# The Digital Impact of Neurosurgery Awareness Month: Retrospective Infodemiology Study

**DOI:** 10.2196/44754

**Published:** 2023-05-08

**Authors:** Kashish Malhotra, Mert Marcel Dagli, Gabrielle Santangelo, Connor Wathen, Yohannes Ghenbot, Kashish Goyal, Ashvind Bawa, Ali K Ozturk, William C Welch

**Affiliations:** 1 Department of Surgery Dayanand Medical College and Hospital Ludhiana India; 2 Department of Neurosurgery Perelman School of Medicine University of Pennsylvania Philadelphia, PA United States

**Keywords:** #NeurosurgeryAwarenessMonth, #Neurosurgery, Neurosurgery Awareness Month, neurosurgery, neural, neuro, health care awareness event, health care, awareness, infodemiology, social media, campaign, neuroscience, neurological, sentiment, public opinion, Google Trends, tweet, Twitter, brain, cognition, cognitive, machine learning algorithm, network analysis, digital media, sentiment analysis, node, Sentiment Viz, scatterplot, circumplex model

## Abstract

**Background:**

Neurosurgery Awareness Month (August) was initiated by the American Association of Neurological Surgeons with the aim of bringing neurological conditions to the forefront and educating the public about these conditions. Digital media is an important tool for disseminating information and connecting with influencers, general public, and other stakeholders. Hence, it is crucial to understand the impact of awareness campaigns such as Neurosurgery Awareness Month to optimize resource allocation, quantify the efficiency and reach of these initiatives, and identify areas for improvement.

**Objective:**

The purpose of our study was to examine the digital impact of Neurosurgery Awareness Month globally and identify areas for further improvement.

**Methods:**

We used 4 social media (Twitter) assessment tools (Sprout Social, SocioViz, Sentiment Viz, and Symplur) and Google Trends to extract data using various search queries. Using regression analysis, trends were studied in the total number of tweets posted in August between 2014 and 2022. Two search queries were used in this analysis: one specifically targeting tweets related to Neurosurgery Awareness Month and the other isolating all neurosurgery-related posts. Total impressions and top influencers for #neurosurgery were calculated using Symplur’s machine learning algorithm. To study the context of the tweets, we used SocioViz to isolate the top 100 popular hashtags, keywords, and collaborations between influencers. Network analysis was performed to illustrate the interactions and connections within the digital media environment using ForceAtlas2 model. Sentiment analysis was done to study the underlying emotion of the tweets. Google Trends was used to study the global search interest by studying relative search volume data.

**Results:**

A total of 10,007 users were identified as tweeting about neurosurgery during Neurosurgery Awareness Month using the “#neurosurgery” hashtag. These tweets generated over 29.14 million impressions globally. Of the top 10 most influential users, 5 were faculty neurosurgeons at US university hospitals. Other influential users included notable organizations and journals in the field of neurosurgery. The network analysis of the top 100 influencers showed a collaboration rate of 81%. However, only 1.6% of the total neurosurgery tweets were advocating about neurosurgery awareness during Neurosurgery Awareness Month, and only 13 tweets were posted by verified users using the #neurosurgeryawarenessmonth hashtag. The sentiment analysis revealed that the majority of the tweets about Neurosurgery Awareness Month were pleasant with subdued emotion.

**Conclusions:**

The global digital impact of Neurosurgery Awareness Month is nascent, and support from other international organizations and neurosurgical influencers is needed to yield a significant digital reach. Increasing collaboration and involvement from underrepresented communities may help to increase the global reach. By better understanding the digital impact of Neurosurgery Awareness Month, future health care awareness campaigns can be optimized to increase global awareness of neurosurgery and the challenges facing the field.

## Introduction

To advocate awareness for neurosurgery, Neurosurgery Awareness Month was initiated by the American Association of Neurological Surgeons (AANS) [1]. Bringing neurological conditions to the forefront and sharing knowledge to enlighten the public, Neurosurgery Awareness Month is observed during the month of August every year. AANS and Neurological Research Foundation advocate the usage of “#NeurosurgeryAwarrenessMonth” for posts related to Neurosurgery Awareness Month on digital media platforms [2,3]. With digital media being one of the key tools to disseminate information, connect with neurosurgeon influencers, and share opinions, it is crucial to understand the impact of awareness months on these platforms to potentially quantify their efficiency and optimize resource allocation [4,5].

Digital media offers a potentially free global reach to increase academic, social, and clinical impact [4,5]. Due to its scalability, it can attract a considerably greater audience than a physical campaign could ever achieve [6,7]. This is further highlighted by an international report, according to which there are 4.7 billion social media users in the world as of July 2022, which equates to 59% of the total global population [8]. Although some health awareness events in other specialties have shown an increased activity of the public in the past, others did not [9-11].

Additionally, health care awareness months have become increasingly prevalent in recent years [12]. In general, these events are designed to bring attention to a specific health condition or issue and encourage individuals to take action toward prevention or management. Although awareness is an important first step, it is not sufficient on its own to create meaningful change. Health care professionals are key players in shaping the future of health care, and as such, it is essential that their understanding and engagement with these awareness months is explored. Despite the massive surge in health care awareness events, there is a lack of standardization and research on the impact of these events on interpersonal, community, and socioeconomic factors, which would justify resource allocation to respective events.

As such, this study aims to fill this gap by examining the impact of a national awareness month (eg, Neurosurgery Awareness Month). By doing so, this study aims to add to the literature that current and future leaders and researchers can use to navigate trends of the web of the future and determine a limited number of digital impact variables for Neurosurgery Awareness Month to get an estimate of its overall global reach and the context of the posts about neurosurgery as well as highlight areas for further improvement.

## Methods

To assess the digital impact of Neurosurgery Awareness Month, we have executed our methodology, which has already successfully generated insight into other health care awareness events [13,14].

### Data Collection

Firstly, we extracted the total number of tweets posted in the month of August between 2014 and 2022 with 2 search queries, using Twitter application programming interface through Sprout Social [15]. Our first search query was specific to Neurosurgery Awareness Month, “neurosurgery awareness month OR #neurosurgeryawarenessmonth OR neurosurgery awareness OR #neurosurgeryawareness” (search query 1). Our second broad search query, “neurosurgery OR #neurosurgery” (search query 2), was used to isolate an overview of neurosurgical posts during the same time frame and was intended as a comparison. The data were collected prior to Twitter acquisition.

### Data Analysis

Extracted results were used to perform appropriate regression analysis to look for overall trends. Total impressions were calculated for #neurosurgery, using Symplurs’s machine learning algorithm [16]. The tweets of each participant and their number of followers were used as input, hypothesizing the maximum number of users who could have been directly reached.

Then, to further shed light on the context of the posts, analysis of both real-time recent and most popular tweets was performed using SocioViz to isolate the Top 100 popular hashtags, keywords, and collaboration between influencers [17]. The search query (neurosurgery OR #neurosurgery) was used as input on the last day of this awareness event to isolate the most popular and recent tweets. Network analysis was performed and illustrated to further highlight impact, reach, and interaction in the digital media environment. Each hashtag is represented as a node. Nodes were linked with each other if there were interactions between them. The size of the nodes was modeled proportional to the number of retweets and mentions received indicating its impact. Different colors were used to differentiate clusters of arguments that were in close proximity to each other. ForceAtlas2’s model was used to show clusters, as it simulates a physical system to spatialize a network. Nodes repulse each other like charged particles, while edges attract their nodes like springs [18]. These forces create a movement that converges to a balanced state and this final configuration helps in the interpretation of the data. Central gravity, spring length, spring constant, gravitational force, and damping were set at 0.3, 95, 0.04, –2000, and 0.09, respectively [18,19].

The search query (neurosurgery OR #neurosurgery) was used to perform sentiment analysis, using Sentiment Viz on the last day of the Neurosurgery Awareness Month for recent tweets. It allows for contextual identification and extraction of subjective information to highlight social sentiment rather than using simplistic measures such as positive, negative, or neutral and has been previously studied as one of the best options in analyzing sentiment of tweets [20]. An emotional scatterplot was formulated based on the circumplex model of affect, with pleasure and arousal on its horizontal and vertical axes, respectively [21-23]. The spatial distribution of the tweets encapsulated their overall sentiment. Each tweet was shown as a circle positioned by “sentiment,” an estimate of the emotion contained in the tweet’s text. Each circle’s color, brightness, size, and transparency visualized different details about the sentiment of its tweet. Colors depicted overall valence or pleasure of a tweet. Pleasant tweets were depicted in green and unpleasant tweets in blue. Active tweets were brighter, which represented the overall arousal of the tweets. Subdued tweets were darker. A circle with larger size depicted a more confident estimate of the tweet’s sentiment. Additionally, transparency was the second measure of confidence in sentiment estimation, where less transparent tweets represented a more confident estimate [21].

Google Trends web search analysis was performed for Neurosurgery Awareness Month from January 2014 to December 2022 for all categories [24]. The worldwide demographic trends were studied using normalized measure of search term by relative search popularity in the set time range [25]. To include all the posts and users globally, we did not set any language or geographical preferences in any of our search queries and included data sets from low search volume regions.

### Ethical Considerations

The use of tweets for research has sparked considerable debate in the academic literature, with many studies indicating that such use is not subject to ethics approval [26,27]. This is due to the fact that tweets posted on Twitter are considered to be in the public domain, and thus, do not fall under the same ethical considerations as human-participant research. We did not seek institutional ethics approval for our study because our data collection was limited to publicly available tweets. To ensure Twitter users’ anonymity, we removed all personal identifying information from the collected data. No human or animal participants were involved. No patient data were collected.

## Results

### Overview

Using our search query, a total of 10,007 users were identified during Neurosurgery Awareness Month using the “#neurosurgery” hashtag that fetched over 29.14 million impressions globally. Of the top 10 most influential users, 5 were US-based neurosurgeons currently working as faculty at various US university hospitals. The rest of the most influential accounts were of notable neurosurgery organizations and journals. Notably, a bot account was one of the top Twitter accounts fetching the highest impressions for #neurosurgery in Neurosurgery Awareness Month.

### Trends

**Figure 1:**
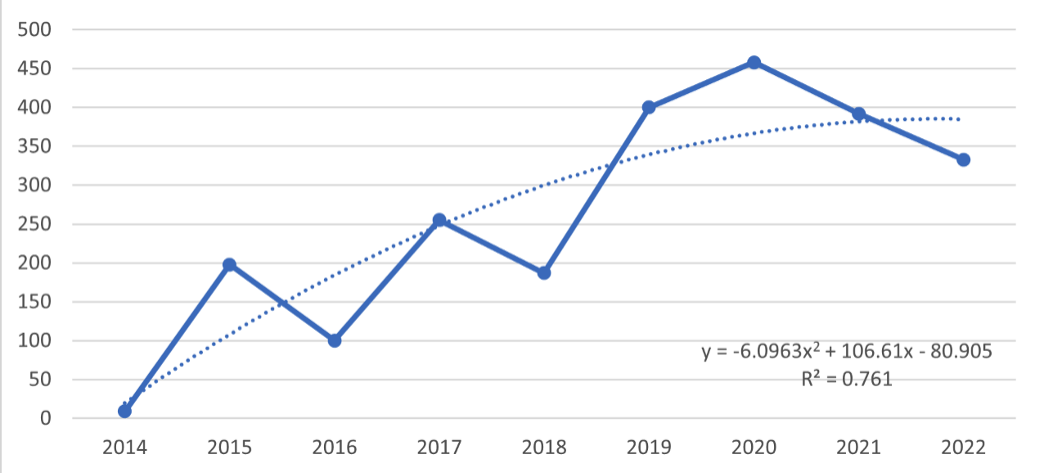
Total tweets specific to Neurosurgery Awareness Month using the search query “neurosurgery awareness month OR #neurosurgeryawarenessmonth OR neurosurgery awareness OR #neurosurgeryawareness” in the month of August from 2014 to 2022.

**Figure 2:**
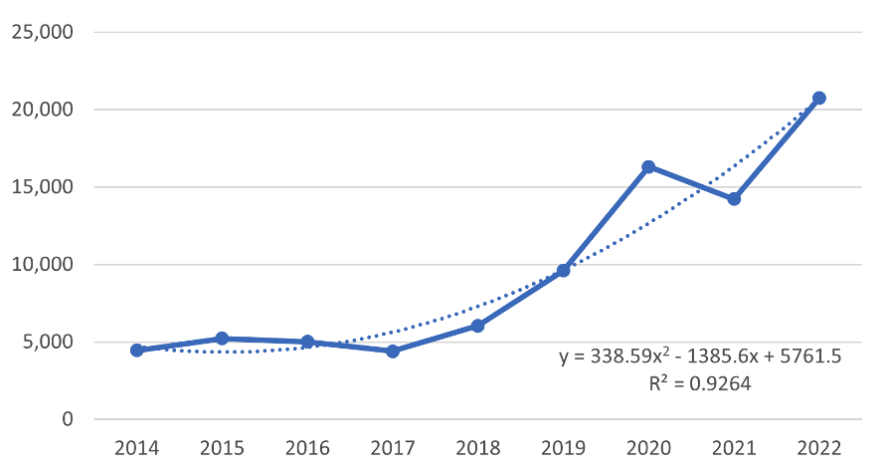
Total tweets about neurosurgery using the search query “neurosurgery OR #neurosurgery” in the month of August from 2014 to 2022.

Retrospective analysis from 2014 to 2022 showed that the highest yearly percentage increase in #neurosurgery tweets (search query 2) of 70% was seen in 2020. Comparing our 2 search queries, 2022 had the highest number of neurosurgery tweets (search query 2), but Neurosurgery Awareness Month–specific hashtags or keywords (search query 1) paled in comparison and did not show an increasing trend (Figure 1 and Figure 2). Only 1.6% of the total neurosurgery tweets were actually advocating about Neurosurgery Awareness Month in August 2022. Only 13 tweets were posted by verified users using the #neurosurgeryawarenessmonth hashtag. Twitter gives verified badge to those influential accounts that are notable, authentic, and active [28].

### Network Analysis

**Figure 3:**
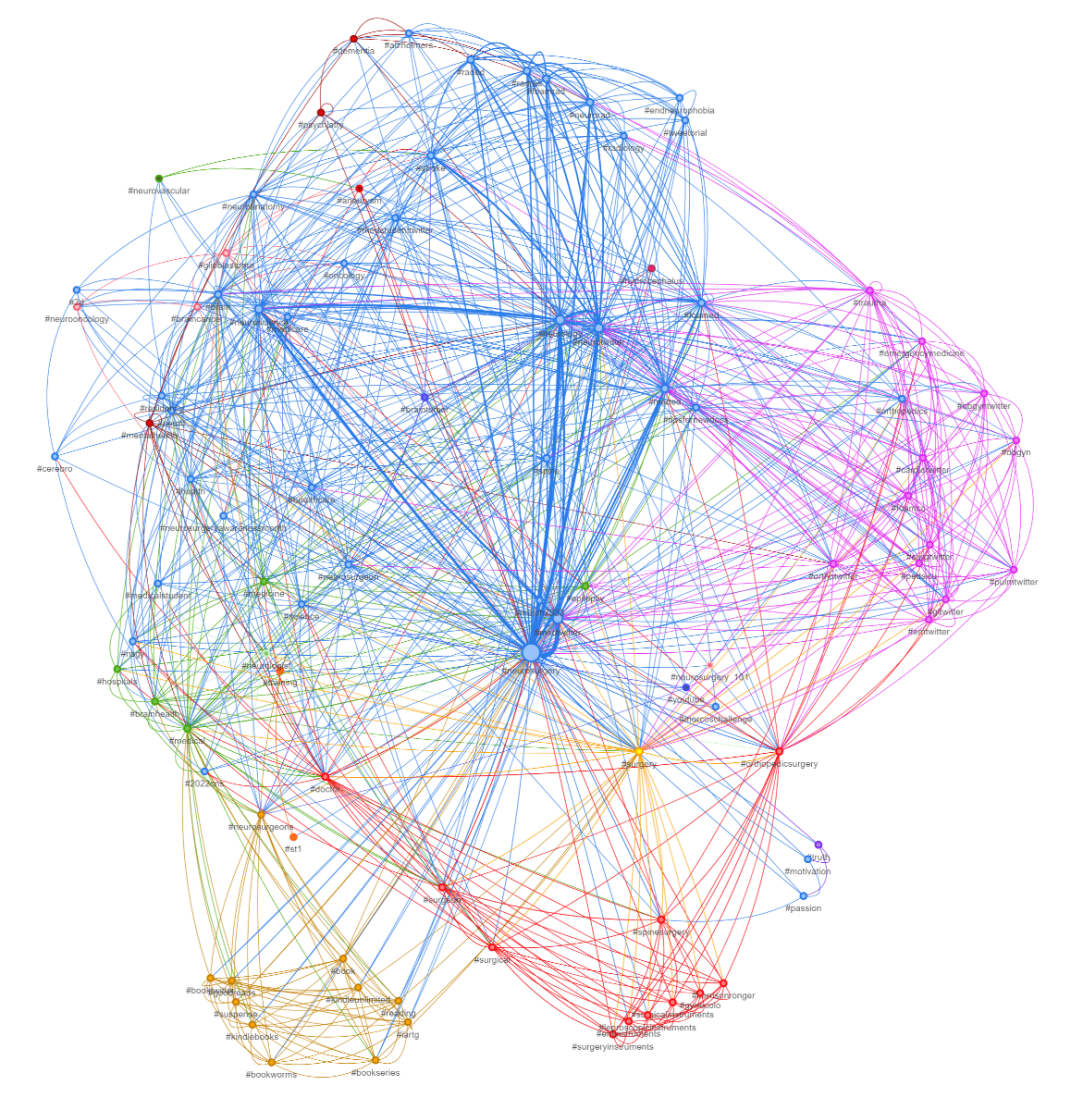
Neurosurgery Awareness Month social network analysis for the search query “neurosurgery OR #neurosurgery”.

**Figure 4:**
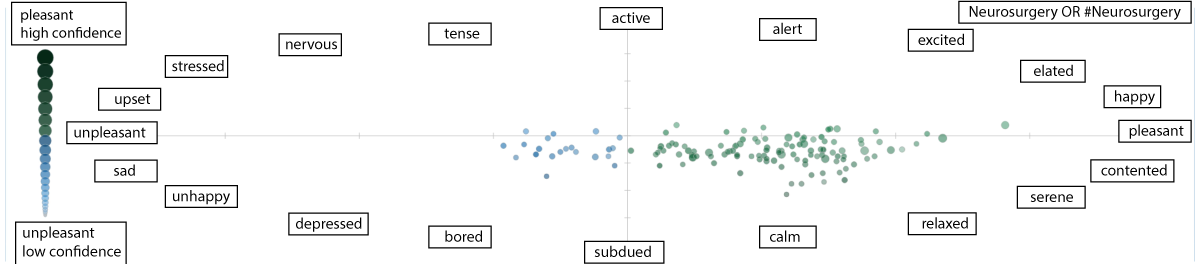
Neurosurgery Awareness Month sentiment analysis for the search query “neurosurgery OR #neurosurgery".

Network analysis of the top 100 influencers, showed a collaboration rate of 81%. The top 3 keywords were “neurosurgery,” “brain,” and “review.” The 3 most used hashtags were #neurosurgery, #medtwitter, and #neurology, followed by other similar hashtags like #neurotwitter, #neuroscience, #meded, #neurorad, #radres, #foamed, and #surgtwitter, as shown in Figure 3. Although the conversations related to neurosurgery and allied specialties like neuropsychiatry, neuro-oncology, and neuroradiology constituted most of the tweets, other themes related to surgery, orthopedics, books, mental health, and medical students were also noticed. The sentiment analysis of the recent neurosurgery tweets showed broadly positive sentiment with subdued emotion (Figure 4).

### Google Trends

**Figure 5:**
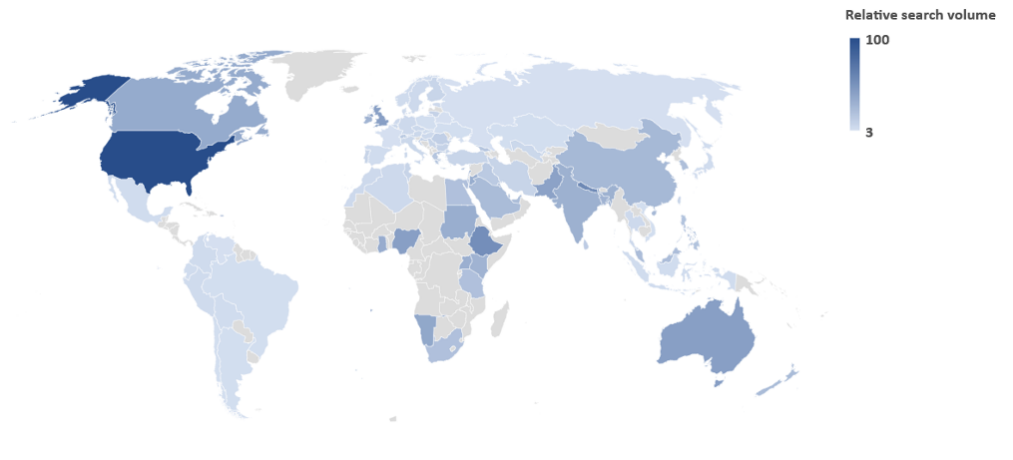
Web search trends for the search term “neurosurgery” from January 2014 to December 2022 on Google Trends.

Due to too little search volume of the Neurosurgery Awareness Month, no significant data could be retrieved from Google Trends for the search term “neurosurgery awareness month.” However, when the data were extracted for the search term “neurosurgery,” significant search volume was noted from the American, European, Australian, and the South Asian regions, with limited involvement from several countries of the African continent and Central Asian countries (Figure 5).

## Discussion

### Principal Findings

Though the efforts of the AANS should be commended for initiating this awareness campaign, the global digital impact of Neurosurgery Awareness Month is nascent, and support from other national and international organizations and stakeholders is needed to yield a significant digital reach. Although there are over 200 health awareness days, weeks, and months recognized on the US National Observances calendar, Neurosurgery Awareness Month is not yet listed [29]. Our analysis showed that Neurosurgery Awareness Month–specific hashtags and keywords reached a very limited audience. In 2022, only 1.6% of total neurosurgery-related tweets could be identified as Neurosurgery Awareness Month–related. We found that tweets related to neurosurgery have increased significantly since 2020; however, the use of the #neurosurgeryawarenessmonth hashtag has been declining. Similar findings were seen in an article evaluating the impact of deep vein thrombosis (DVT) month, where DVT awareness–specific tweets were less than 3% of the total DVT tweets [13].

A strength of the Neurosurgery Awareness Month is that it is spearheaded by the AANS, unlike many awareness campaigns that are supported by nonmedical community members. However, Neurosurgery Awareness Month still had limited digital reach, which is contrasting to the findings of World Hypertension Day, which is also supported by various international medical organizations and amassed over 250 million impressions [14]. This highlights the unmatched potential of social media that can be effectively used to promote Neurosurgery Awareness Month.

Our study showed collaboration rate of 81% among top influencers, with several neurosurgeons and reputable organizations driving the conversations about Neurosurgery Awareness Month. A recent analysis of the effect of Breast Cancer Awareness Month through Twitter demonstrated that the most effective tweets were from celebrities and organizations that promoted early detection and fundraisers and that the majority of social media engagement occurred in the first few days of the month [30]. Similar findings were noted when the digital impact of Polycystic Awareness Month was studied [31].

Other than just optimizing that a goal-directed message from Neurosurgery Awareness Month is available and supported on social media, it is crucial to recognize disparities in neurosurgery and highlight the concepts of equity in global surgery and global neurosurgery [32,33]. In our study, neurosurgery influencers and search interest were mostly seen from the United States. This is consistent with a recent study identifying the top 100 Twitter neurosurgery influencers [34]. This reflects the need to prioritize and highlight issues pertaining to underrepresented and underprivileged communities to promote involvement outside of the United States, United Kingdom, and Canada [35]. Limited global representation has previously been noticed in Hernia Awareness Month [11]. Further studies may consider exploring the perspectives of influencers and understanding the utility of social media to relay medical information thematically [36].

Although #NeurosurgeryAwarenessMonth had limited reach, considerations such as adopting an abbreviation to be used across digital platforms for the Neurosurgery Awareness Month could optimize the branding of the event and increase search engine optimization for studying which posts were directly relating to the awareness month [37,38]. Involving general public, especially from the non-US–based neurosurgical community, will improve the overall impact and global reach of Neurosurgery Awareness Month. By increasing collaboration, this campaign has the potential to grow our global community, involving various stakeholders, such as doctors, general public, patients, and policy makers, for cumulative reach.

Neurosurgery Awareness Month has evolved over the years; its initial focus was highlighting neurosurgical safety issues and then expanded to elucidating specific neurosurgeons and how they care for their patients [2], ensuring that the focus and intention of the Neurosurgery Awareness Month is paramount in its future success with goal-directed content. This includes the content directed to the general public and to those within the global neurosurgery community. With a clear intention, evaluation of effectiveness in changing behaviors, improving knowledge, or changing perceptions becomes possible [12]. However, before an awareness campaign can effect change in a public health domain, awareness of the campaign must exist [39]. Finally, the effectiveness in delivering the desired message to the intended population should be studied for Neurosurgery Awareness Month as has been with other disease awareness campaigns [12]. It has been suggested that health awareness days may actually place the responsibility for adverse health outcomes on the individual’s lack of knowledge. Ensuring that content is sensitive to the limitations of its audience is crucial. Another important consideration is that this campaign, with possible future recognition from legislation, may increase policy makers’ awareness to neurosurgical issues [12].

### Limitations

The study’s reliance on social media data, which may not accurately represent the full scope of global interest in Neurosurgery Awareness Month, is one limitation. Another consideration is that our study’s findings are limited to a single health care awareness event and may not be generalizable to other awareness campaigns or neurosurgery in general. Neurosurgery Awareness Month’s specific conditions, goals, and strategies may not be representative of other awareness efforts, and more research is needed to understand the broader impact of such campaigns on public knowledge and attitudes toward health care. Moreover, the study’s data analysis techniques may have introduced bias into the results. For example, the use of only 4 social media assessment tools may have resulted in the exclusion of important data or perspectives. As a result, the findings of the study may not accurately reflect the impact of Neurosurgery Awareness Month on social media, and further research using more comprehensive and objective methods may be needed to fully understand the digital impact of such campaigns. Finally, the study focuses on English-language social media data, which may exclude important perspectives and experiences from non–English-speaking individuals. As a result, the findings of the study may not accurately reflect the global impact of Neurosurgery Awareness Month. Further research using a more diverse range of language data may be needed to more fully understand the global impact of Neurosurgery Awareness Month.

### Conclusions

Neurosurgery Awareness Month was initiated by the AANS to raise awareness of neurosurgical diseases and connect the global neurosurgical community. The digital impact of Neurosurgery Awareness Month is nascent with limited involvement from several African and Central Asian countries. Although the conversations and sentiments of posts were mostly about neurosurgery and allied specialties with pleasant sentiment, increasing collaboration and involvement from underrepresented communities may help to increase the global reach. Finally, it should be highlighted that there is a major lack of standardized variables that could be used for analysis of health care events and function as the foundation for evidence-based resource allocation optimization, due to a lack of specific aims and missing consensus. Thus, there is a major need for more complex research on this topic, development and improvement of impact analysis protocols, and increased awareness by stakeholders.

## Abbreviations

AANS: American Association of Neurological Surgeons
DVT: deep vein thrombosis

## Data availability

The original contributions presented in the study are included in the paper. Further inquiries can be directed to the corresponding author.
